# Predicting Enantioselectivity via Kinetic Simulations on Gigantic Reaction Path Networks

**DOI:** 10.1021/acscentsci.6c00079

**Published:** 2026-03-30

**Authors:** Yu Harabuchi, Ruben Staub, Min Gao, Nobuya Tsuji, Benjamin List, Alexandre Varnek, Satoshi Maeda

**Affiliations:** † Institute for Chemical Reaction Design and Discovery (WPI-ICReDD), 12810 Hokkaido University, Kita 21, Nishi 10, Kita-ku, Sapporo, Hokkaido 001-0021, Japan; ‡ JST, ERATO Maeda Artificial Intelligence in Chemical Reaction Design and Discovery Project, Kita 10, Nishi 8, Kita-ku, Sapporo, Hokkaido 060-0810, Japan; § Max-Planck-Institut fur Kohlenforschung, Mulheim an der Ruhr 45470, Germany; ∥ Laboratory of Chemoinformatics, UMR 7140, CNRS, University of Strasbourg, 67081 Strasbourg, France; ⊥ Department of Chemistry, Faculty of Science, Hokkaido University, Kita 10, Nishi 8, Kita-ku, Sapporo, Hokkaido 060-0810, Japan

## Abstract

Asymmetric catalysts exert control over the reactivity and enantioselectivity of chemical reactions, often through their large size and highly flexible geometries. Most computational approaches rely on a predefined selectivity-determining step and assume that selectivity follows the Boltzmann distribution of transition-state conformers. Going beyond this conventional framework, we constructed a reaction path network that captures the kinetically accessible regions of the potential energy surface. A delta-learning neural network potential (ΔNNP) was constructed and applied to an asymmetric organocatalyst of the imidodiphosphorimidates family, comprising over 200 atoms. The ΔNNP achieved DFT-level accuracy with GFN2-xTB as the baseline. Using the ΔNNP-based single component-artificial force induced reaction (denoted by NNP/AFIR) method, we constructed a reaction path network containing 48,463 paths. Kinetic simulations based on this network predicted the reaction mechanism and enantioselectivity. Within the network, numerous paths, including asynchronous concerted and stepwise mechanisms, were found to be energetically competitive and thus to contribute to the overall kinetics. Traffic volume analysis further revealed that intermediates with negligible yields can still play important kinetic roles. Overall, the NNP/AFIR approach provides a powerful framework not only for deepening mechanistic understanding but also for accelerating the rational design of asymmetric catalytic systems with high reactivity and enantioselectivity.

## Graphical abstract

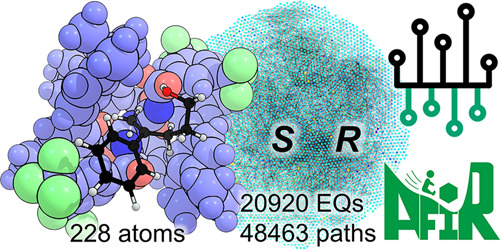

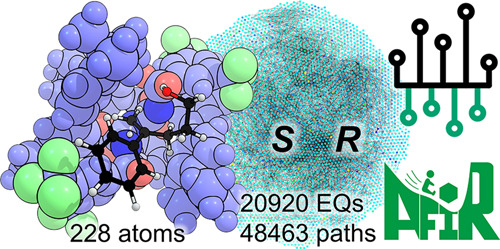

## Introduction

Over the last century, asymmetric catalysis has undergone drastic development, spanning both biocatalysis and artificial molecular catalysis. While the latter, whether or not containing transition metals,^1–3^ initially employed relatively simple molecular scaffolds, recent advances have introduced catalysts with increasing molecular size and flexibility to address challenging stereoselective transformations. As the structural diversity of asymmetric catalysts has expanded, understanding how their large and flexible architectures influence stereochemical outcomes has required increasingly detailed investigation. The imidodiphosphorimidate (IDPi) catalyst developed by the List group exemplifies this trend.^4,5^ Its high acidity and confined microenvironment have enabled a variety of stereoselective transformations, yet detailed mechanistic elucidation remains challenging due to its structural and dynamic complexity. Moreover, IDPi-catalyzed reactions often involve carbocationic intermediates, generating intricate reaction landscapes with multiple competing conformations and mechanistic pathways. These features render intuition-based analysis difficult and motivate the development of more comprehensive computational approaches.

In recent decades, advancements in computational chemistry^6^ have significantly contributed to the mechanistic understanding of homogeneous catalysis and the origins of its selectivity.^7–13^ Density functional theory (DFT) has been applied to characterize the enantioselectivity based on reaction pathways with transition state (TS) structures, enabling analysis of how structural and electronic factors correlate with stereochemical outcomes. These insights have, in turn, inspired the rational design of new catalysts. For catalyst systems with large and flexible molecular frameworks, it has been noted that meaningful evaluation of enantioselectivity typically requires systematic conformational sampling over a broad ensemble of TS geometries. Consequently, diverse computational strategies have been proposed to improve the systematic exploration of the TS conformers.^14–22^ By assuming a Boltzmann distribution of TS conformers, enantioselectivities that compare directly with experimental data can be obtained.

As the number of relevant conformations grows, the associated computational cost increases rapidly, making it challenging to balance computational accuracy with practical feasibility. To reduce computational costs of gradient calculations used in the sampling procedure, multiscale and semiempirical approaches, such as QM/MM^23,24^ and GFN*n*-xTB,^25,26^ have been employed in the initial screening stage. However, these methods often struggle to accurately capture interactions between the unstable reaction center at the TS and surrounding unreactive substituents, which are critical factors influencing enantioselectivity. A commonly used strategy is therefore a multistep protocol in which these low-cost methods are used to generate candidate geometries, followed by reoptimization and energy refinement at higher-level DFT. This hierarchical approach allows the consideration of a broader structural ensemble while maintaining adequate accuracy for mechanistic analysis.

Despite the successful application of these methods to enantioselectivity prediction, significant challenges remain. These methods require a predetermined selectivity-determining step. Moreover, predicting enantioselectivity based on the Boltzmann distribution of TS conformers relies on two assumptions: that the system reaches equilibrium before the selectivity-determining step, and that the reverse reaction has a zero rate constant.^27^ Consequently, approaches that focus on predetermined steps and their TS conformer ensembles face limitations when the reaction mechanism is unknown, when multiple mechanistic pathways contribute simultaneously, when the selectivity-determining step occurs before pre-equilibrium is established, or when racemization occurs via reverse steps.

In such cases, the analysis of asymmetric catalysis requires methods that can explore diverse reaction paths and enable kinetic simulation without relying on assumptions. Consequently, the development of theoretical techniques that combine accuracy with minimal prior specification remains an important objective in the study of complex catalytic systems. Automated reaction-path search methods offer a powerful means for unbiased and systematic exploration of possible chemical transformations.^28–34^ However, achieving sufficiently comprehensive sampling of both conformational space and chemical bond-pattern space simultaneously remains computationally challenging for systems involving large, flexible catalysts of current interest.

Here, we present a solution to this challenge and demonstrate its application to a practical organocatalytic system. The AFIR method,^35,36^ a reaction path search approach generating a reaction path network, has been proposed and successfully applied to predict reaction paths for systems containing 10∼50 atoms at a DFT level.^37,38^ Because the AFIR method explores a broad region of the potential energy surface, it can identify reaction mechanisms without relying on prior assumptions about the target system. Moreover, the combination of on-the-fly kinetic simulations^39^ with the AFIR method enables systematic exploration of kinetically accessible regions, allowing the construction of reaction path networks that represent the underlying reaction kinetics. In particular, the rate constant matrix contraction (RCMC) method,^40^ a kinetic simulation method, provides an efficient and numerically stable framework for predicting product yields under specified reaction conditions.^41^ This enabled the prediction of selectivity and reactivity based on large reaction path networks containing over 10,000 reaction paths. On the other hand, the reaction path searches remain computationally demanding for large systems consisting of more than 50 atoms, particularly when relying on DFT calculations. This situation has been substantially improved by frameworks that combine the neural network potentials (NNPs) with a Δ-learning approach^42–44^ and the AFIR method (referred to as the NNP/AFIR method). This combined scheme enables efficient construction of accurate reaction path networks, including equilibrium structures (EQs) as well as TS regions.^45,46^

In this study, the NNP/AFIR method was used to investigate an asymmetric hydroalkoxylation reaction of alkene catalyzed by the IDPi, involving a total of 228 atoms and proposed to proceed via carbocationic intermediates (Figure 1).^47^ This reaction therefore serves as a stringent test case for evaluating the ability of NNP/AFIR to resolve mechanistic complexity in realistic asymmetric catalysis. Experimentally, this reaction afforded an 84% yield and high enantioselectivity (*S*:*R* = 98.5:1.5) after 2 days at 60 °C in cyclohexane. Within this framework, the NNP/AFIR calculations generated a reaction path network consisting of 20,920 equilibrium structures (EQs) and 48,463 reaction paths. Kinetic simulations based on this network reproduced the enantioselectivity, establishing NNP/AFIR as a generalizable framework for tackling such mechanistically complicated, conformationally flexible systems.

**Figure 1:**
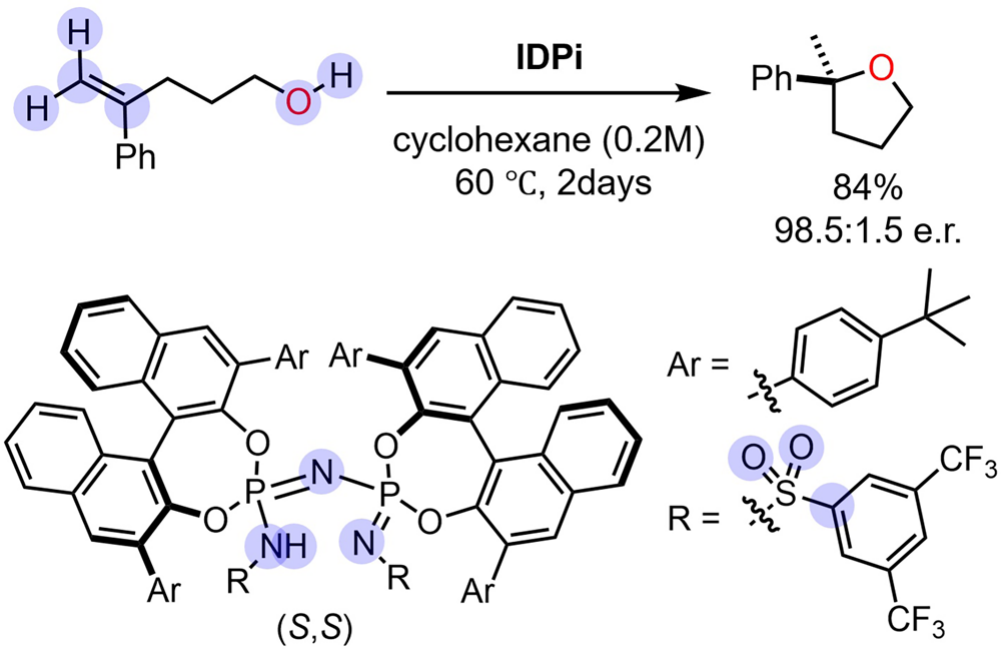
Asymmetric hydroalkoxylation reaction of alkene with the IDPi reported in the previous study.^47^ Experimental condition, reaction yield, and enantiomer ratio are indicated. The target atoms in the AFIR search are indicated by the blue shadows.

## Results and Discussion

Table 1 summarizes the size of the database and reaction path networks for 9 training iterations. The table includes the job type (type), the number of computed AFIR paths (*n*PATH), the number of EQs and TSs in the network (*n*EQ, *n*TS), the number of computed gradients (force), the model accuracy achieved (acc.), and the number of converged DFT calculations added to the database (train add). As shown in Table 1, the database and NNP training start with a small initial database and gradually increase the database size from 400, 1600, 1600, 1600, 3200, to 25600. Details are discussed in Computational details and in SI1.

**Table 1:** Reaction Path Search Settings, Size of Reaction Path Network, Number of Gradient Calculations, and Size of DFT Database for Iterations 1–9^a^

	type	nPATH	γ^b^	nEQ	nTS	force	acc.^a^	train add
1	AFIR	400	300	707	1016	1.3 M	40.2	1714
2	AFIR	1600	300	5015	9874	10 M	11.5	14858
3	AFIR	1600	300	5011	10742	14 M	4.8	15734
4	AFIR	1600	300	5739	11915	16 M	2.2	17631
5	AFIR	3200	300	14922	37063	37 M	1.9	51890
6	AFIR	25600	200	14834	39193	45 M	-	-
7	Repath	-	-	18193	42224	69 M	1.5	60301
8	Repath	-	-	20296	48552	81 M	-	-
9	Hessian			20920	48463	201 M	1.2^c^	-

a The energy error of each NNP model with DFT is shown in kJ/mol.

b The mean absolute error (MAE) for the NNP model is shown in kJ/mol.

c The model collision energy for the AFIR search is shown in kJ/mol.

d Geometries randomly selected at a rate of 1/50 from all EQs and TSs (i.e., 418 EQs and 970 TSs) were computed at the same DFT level to evaluate the NNP model accuracy for structures with energies between 0 and 400 kJ/mol. These data are not used to train the NNP model.

Figure 2 shows a parity plot comparing the NNP model with DFT calculations for structures randomly sampled at a rate of 1/50 from all EQs and TSs at iteration 9 (i.e., 418 EQs and 970 TSs). The results demonstrate that the final NNP model achieves an accuracy comparable to that of DFT. As summarized in Table 1, the number of data points increased from 0 to 162,128 through the iterations, while the mean absolute error (MAE) of the NNP decreased from 40.2 kJ/mol (iteration 1) to 1.2 kJ/mol (iteration 9), highlighting the efficiency of the iterative training procedure.

**Figure 2:**
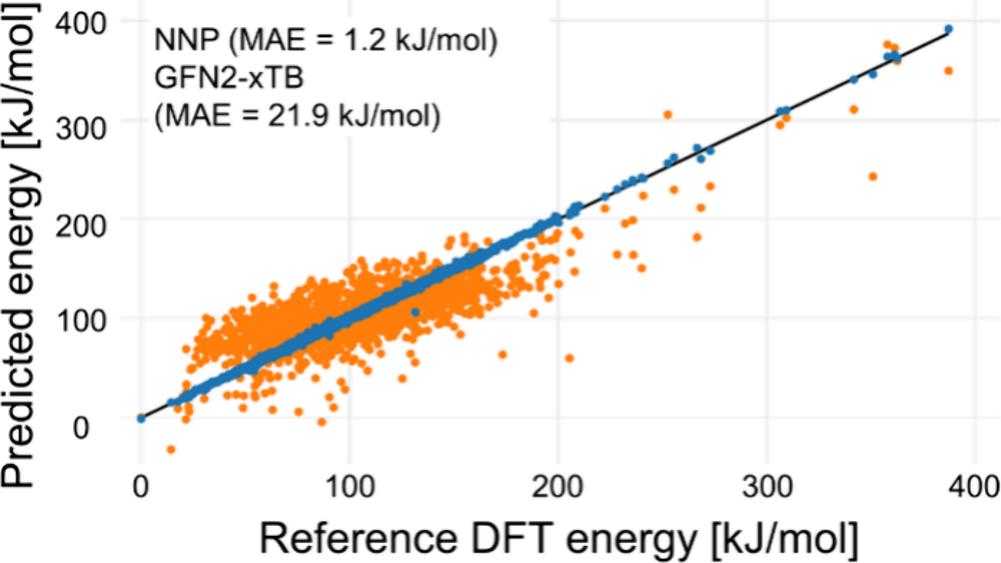
Accuracy of the energies obtained from the NNP at the final (the 9th) iteration. The reference energy is set to that of the lowest-energy geometry.

The yield was estimated to be 99.26% and enantiomer ratio (*S*:*R* = 99.95:0.05), which is in good qualitative agreement with the experimental value.^47^ Note that a direct comparison of absolute yield values between computation and experiment (i.e., 99.26% vs 84%) would not be straightforward, as some product loss inevitably occurs during postreaction workup. Although the calculation overestimated the S selectivity, the barrier gap between the (*S*) and (*R*) formations estimated from the computed yield ratio is 17.4 kJ/mol, which is sufficiently close to the experimental value of 11.6 kJ/mol, with the deviation approaching the typical chemical accuracy range of ∼4.2 kJ/mol. Improvements may be expected by using larger basis sets (e.g., triple-ζ basis sets) and higher-level electronic structure methods such as coupled cluster. This result demonstrates that the kinetic simulation based on the reaction path network predicts the reactivity and selectivity of the asymmetric organocatalytic reaction without requiring prior knowledge specific to the target reaction. Figure 3 summarizes the main routes extracted from the entire reaction path network. EQs are grouped according to their bonding patterns and the absolute configuration of the product region. Only the EQ with the lowest Gibbs energy in each group and the lowest-energy route connecting different groups are shown. This simplified representation provides an overview of the reaction mechanism, with each group comprising hundreds or more conformers. Note that all conformers and their corresponding TSs were included in the kinetic analysis, and all the EQ and TS geometries are provided via the Zenodo repository.

**Figure 3:**
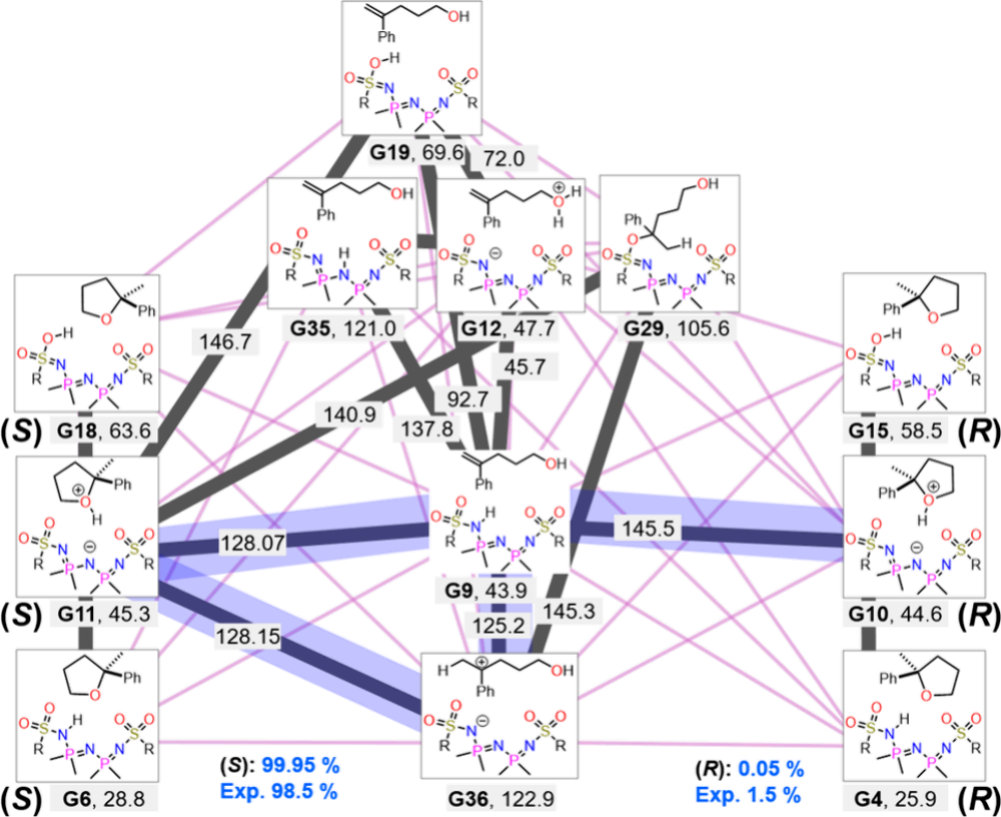
Extracted reaction path network by grouping EQs based on their bonding patterns and the absolute configuration of the product region. The Gibbs energy is shown in kJ/mol relative to the most stable geometry in the network. All EQs are classified into 74 groups based on bonding patterns and stereo conformation at the reaction site, as described in SI1. The groups are sorted by Gibbs energy and made available via the Zenodo repository. The TS energies lower than 150.0 kJ/mol are highlighted in the gray lines. The lines between each group indicate the TSs between them. The calculated reaction yields and enantiomer ratio are shown in blue numbers.

It was shown that there are three types of reactants and products, which are classified based on the position of the proton: (1) species protonated at the sulfonyl group, (2) species protonated at the nitrogen atom of the IDPi center, and (3) protonated at the oxygen atom of reactants/products. When the alkene is protonated, the carbocation intermediate (G36) is formed. The intermediate, G29, with the formed carbocation C–O (O of sulfonyl group) bond is also obtained. Multiple paths from reactants to products were identified within the network.

In the network, two major reaction paths were observed. The first one is an asynchronous concerted path, where the reactant converts directly into the product in a single step (G9-G11; G9-G10). The second is a stepwise path (G9-G36-G11; G9-G36-G4), in which the reaction proceeds through a metastable carbocation intermediate before product formation. Interestingly, the former mechanism is favorable for the formation of the (*R*)-product, while, in the case of (*S*)-product, both mechanisms give the TSs with very similar energies, indicating competition between the two mechanisms. The TS of the asynchronous concerted path corresponds to the protonation step, whereas the rate-determining step of the stepwise mechanism corresponds to the cyclization from a carbocation intermediate. Thus, the two mechanisms involve fundamentally different processes.

The most stable TSs for the asynchronous concerted and stepwise reaction paths leading to the products were compared. The TS structures of the concerted path are similar to the ones reported by Bistoni et al.^48^ It is shown that two π–π interactions are observed between the arylsulfonyl group and the BINOL backbone in the path leading to both enantiomers of the product.

Moreover, the reaction path network highlights the importance of substrate prearrangement. Substrate prearrangement has also been recognized as playing an important role in systems involving artificial enzymes and biomimetic catalysts.^49^ Figure 4 shows a comparison of the geometries of reactant G9 and carbocation G36. The most stable conformers of both species possess relatively linear arrangements, stabilized by two hydrogen bonds between the −OH group and the catalytic center. In these thermodynamically preferred structures, the distance between the reacting C and O atoms is relatively long (∼3.9 Å as shown in Figure 4a), and the geometry connected to the lowest TS for carbocation formation exhibits an even longer separation, ∼4.2 Å, as shown in Figure 4b.

**Figure 4:**
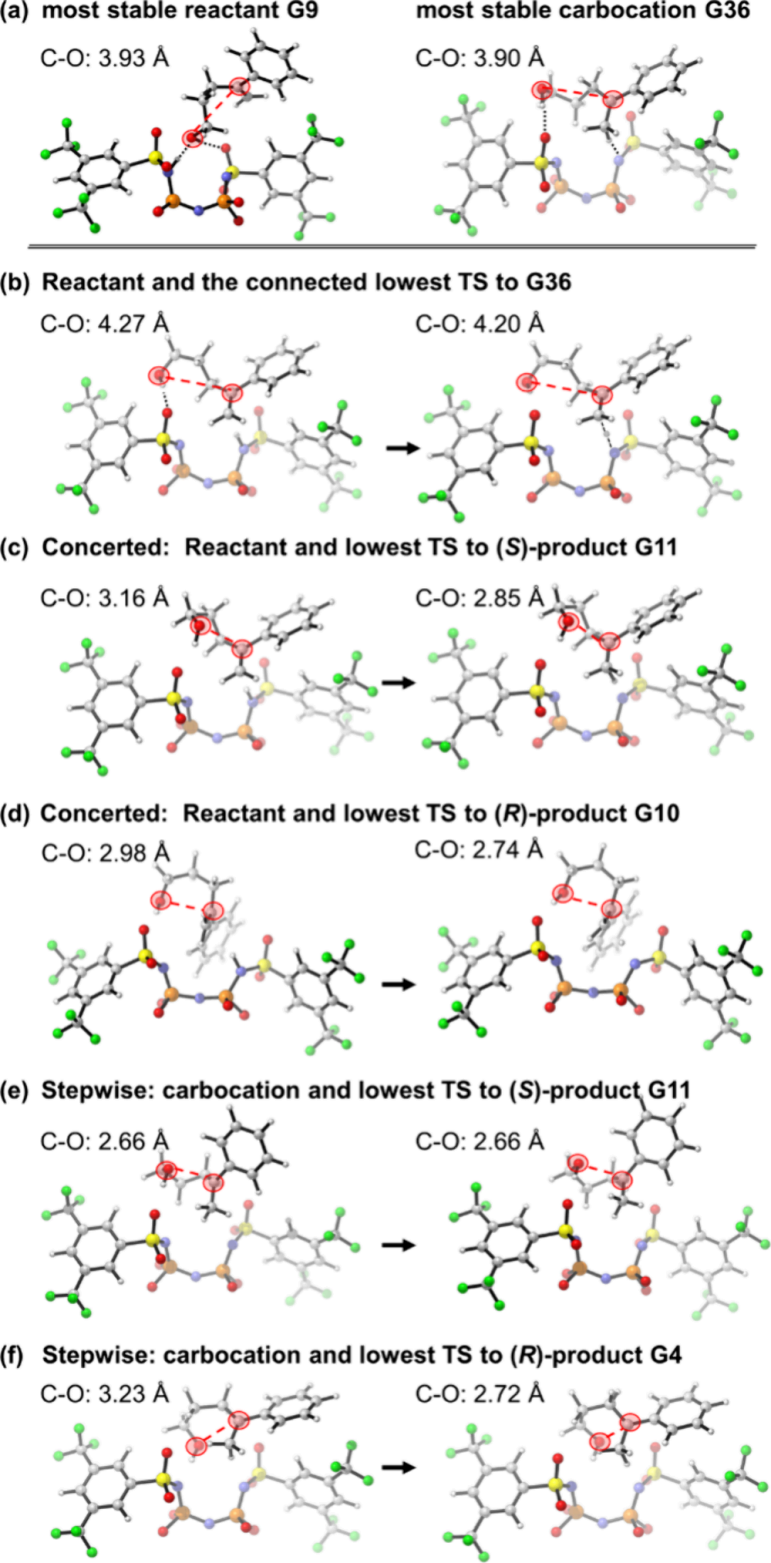
Comparison of the geometries of reactant G9 and carbocation G36. (a) Most stable geometries of G9 and G36. (b) Lowest-energy TS for G36 formation and the TS linked to G9. (c) Lowest concerted TS to (*S*)-product G11 and the TS connected to G9. (d) Lowest concerted TS to (*R*)-product G10 and the TS connected to G9. (e) Lowest TS for the stepwise path from G36 to (*S*)-product G11 and the TS connected to G36. (f) Lowest TS for the stepwise path from G36 to (*R*)-product G4 and the TS connected to G36. Geometries were plotted by CYLview, 1.0b.^50^

For the asynchronous concerted mechanism, however, the reactant geometries that connect to the lowest-energy TSs for the (*S*)- and (*R*)- products possess more compact arrangements. In these conformers, the reacting atoms are brought into close proximity, and the –OH group rotates toward the reacting alkene as shown in Figure 4c and d, thereby inducing spontaneous cyclization following protonation. For the stepwise mechanism, the linear type reactant is first protonated to form a linear carbocation intermediate. This intermediate must then fold into a compact geometry before the final cyclization takes place. These structural differences clearly demonstrate that prearrangement is crucial for both mechanisms, highlighting the necessity of an exhaustive search when elucidating reaction mechanisms.

During the kinetic simulations, the traffic volume,^51,52^ defined as the sum of the population influx to and outflow from each EQ, was computed. The traffic volume for each group is shown in Figure 5. The 12 groups depicted in Figure 3 exhibit relatively high traffic volume values. The number of conformers included in each group reflects the complexity arising from possible geometrical effects. The traffic volume of the protonated reactant G12 is of a similar magnitude of G9, highlighting its importance in the reaction. The traffic volumes of G12, along with the reactant with the proton at different positions (G35: central N; G19: O atom) of the IDPi reaction center, and G29, an intermediate with a formed bond between the C of carbocation and sulfonyl O bond, suggest that these species are kinetically accessible from the reactant region. Traffic volumes were also observed for three types of (*S*)- and (*R*)-products. This situation requires extensive exploration of many conformations and reaction paths during the search.

**Figure 5:**
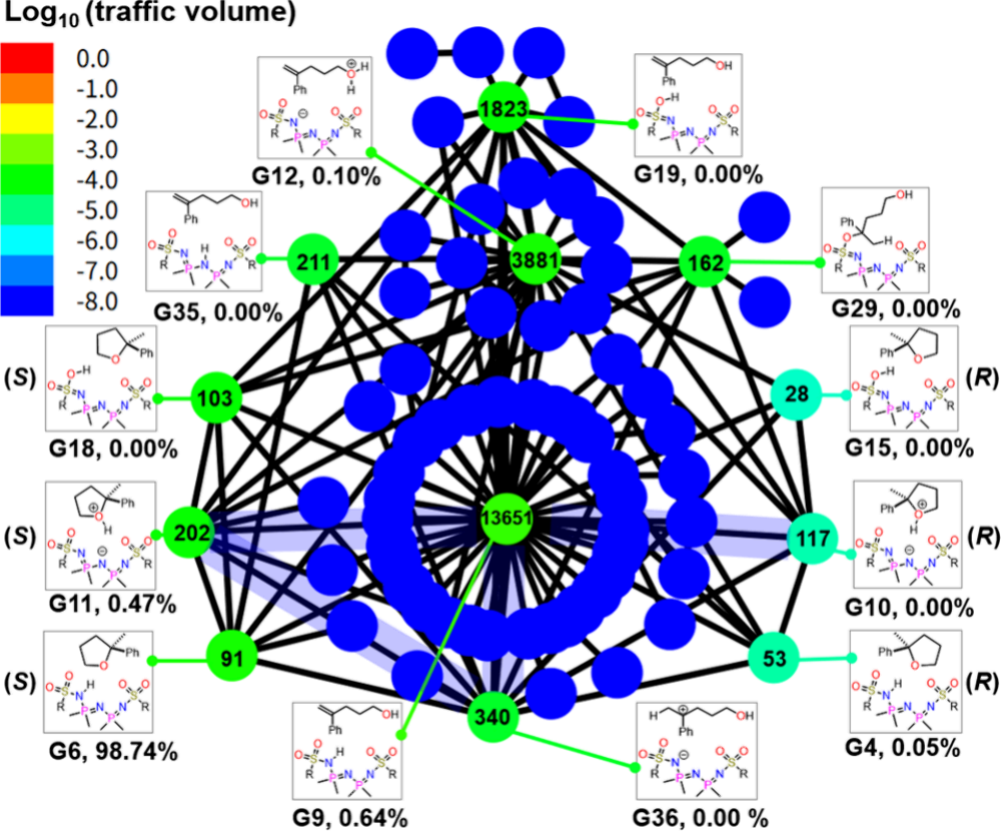
Log_10_ of traffic volumes for groups in the reaction path network. Each cycle indicates one intermediate group. Numbers inside the cycles indicate the number of conformers in each group. The kinetic simulation settings are the same as those used for the yield calculations, and the corresponding reaction yields are also indicated.

The extensive search for many types of reaction paths is computationally demanding (iteration 6 requires 45 million gradient calculations). This study overcomes this situation using the NNP/AFIR method and demonstrates understanding of the asymmetric catalysis without prior expertise in the target system. On the other hand, the generation of 0.06 million DFT data points in the final database creation required the use of the supercomputer Fugaku, utilizing on the order of 30,000 CPU cores. Therefore, the present methodology cannot yet be regarded as routine from the perspective of computational resource requirements. Nevertheless, ongoing advances in universal NNP models^53^ are expected to substantially reduce computational cost, making such comprehensive reaction analyses increasingly accessible. In this context, clarifying the precise relationship between reaction path network analysis and conventional TS sampling strategies remains an important topic for future investigation. Moreover, the reaction examined in this work exhibits very high experimental enantioselectivity and thus represents only one selectivity regime. Extending this framework to reactions with lower enantiomeric excess will provide a more stringent benchmark and further establish the predictive reliability of network-based selectivity analysis.

## Conclusion

In this study, the NNP/AFIR method was employed to generate a reaction path network for an asymmetric catalytic system comprising 228 atoms. A large-scale network of 48,463 reaction paths was constructed with an accuracy comparable to the DFT level. The kinetic simulation based on the obtained reaction path network explained the experimental enantioselectivity. The traffic volume analyses based on kinetic simulations further emphasize the importance of exhaustive reaction path exploration, demonstrating that selectivity-determining channels are highly competing. Furthermore, analyzing catalyst and substrate geometries provides insight into the importance of prearrangement in the target reaction. This approach is expected to expand the frontier of quantum chemical analysis and to contribute significantly to the design of highly functional organocatalysts. The results also highlight the potential of network-based reactivity prediction for practical applications to asymmetric catalyst development. In particular, future studies applying this framework to reactions with both low and high selectivity are expected to further demonstrate its predictive power and advance reaction and catalyst design.

## Computational Details

An NNP model was trained through an iterative training procedure, involving model-powered exploration, DFT validation, and repeated model updates. GFN2-xTB,^26,54^ a semiempirical method, is employed as the baseline of the Δ-learning approach, and the methodology is detailed in our previous work.^45,46^ In this study, all the reaction paths are relaxed using the locally updated planes (LUP) method,^55,56^ and the energy maximum point along the paths is used as a TS geometry during the kinetic analyses. DFT database was generated using Gaussian16 ^57^ at the computational level of ωB97X-D^58^/Def2-SVP, including cyclohexane solvent effects modeled by the polarizable continuum model (PCM) method.^59^ The influence of basis set size on the energy of key intermediate and TS energies was acceptable, as summarized in Table S3. The difference between energies obtained by NNP and ωB97X-D/Def2-SVP is negligible, which indicates the good accuracy of the trained NNP.

In the NNP/AFIR search, 16 atoms from the reactant and catalyst centers were selected as target atoms as described in SI1. To efficiently sample the chemical space, the model collision energy of AFIR was set to 300.0 kJ/mol for iterations 1 to 5 and reduced to 200.0 kJ/mol for iteration 6. During the NNP/AFIR search, a weak universal force was applied to maintain the reactant close to the catalyst center region. This weak force was removed in iterations 7–9, and the reaction paths were relaxed and recalculated (indicated by repath). Gibbs free energies were estimated by harmonic vibrational analysis in iteration 9 by computing the numerical Hessian at EQs and TSs at the experimental temperature 333.15 K. Based on the Gibbs free energies, the RCMC kinetic simulation was performed to predict yields and enantioselectivity. During the RCMC simulation, the energies of several geometries (7 EQs and 10 TSs) were replaced to avoid the contamination of these geometries during the analyses. Reaction path search using the NNP/AFIR method was performed with the developmental version of GRRM.^60^

## Supplementary Material



## Data Availability

All optimized equilibrium (EQ) and transition state (TS) geometries, along with their free energies, are provided in the Zenodo repository (DOI: 10.5281/zenodo.17962664).

## Notes

Details of the computational level (SI1), the target atoms of NNP/AFIR search (Figure S2), functional/basis set dependence (Table S3), and the traffic volume of the intermediate in the reaction path network (Table S4) (PDF)

## References

[1] Noyori R. (2002). Asymmetric
Catalysis: Science and Opportunities (Nobel Lecture). Angew. Chem., Int. Ed..

[2] List B. (2007). Introduction:
Organocatalysis. Chem. Rev..

[3] MacMillan D. W. C. (2008). The
Advent and Development of Organocatalysis. Nature.

[4] Kaib P. S. J., Schreyer L., Lee S., Properzi R., List B. (2016). Extremely
Active Organocatalysts Enable a Highly Enantioselective Addition of
Allyltrimethylsilane to Aldehydes. Angew. Chem.,
Int. Ed..

[5] Schreyer L., Properzi R., List B. (2019). IDPi Catalysis. Angew. Chem., Int. Ed..

[6] Blum V., Asahi R., Autschbach J., Bannwarth C., Bihlmayer G., Blügel S., Burns L. A., Crawford T. D., Dawson W., De Jong W. A., Draxl C., Filippi C., Genovese L., Giannozzi P., Govind N., Hammes-Schiffer S., Hammond J. R., Hourahine B., Jain A., Kanai Y., Kent P. R. C., Larsen A. H., Lehtola S., Li X., Lindh R., Maeda S., Makri N., Moussa J., Nakajima T., Nash J. A., Oliveira M. J. T., Patel P. D., Pizzi G., Pourtois G., Pritchard B. P., Rabani E., Reiher M., Reining L., Ren X., Rossi M., Schlegel H. B., Seriani N., Slipchenko L. V., Thom A., Valeev E. F., Van Troeye B., Visscher L., Vlček V., Werner H.-J., Williams-Young D. B., Windus T. L. (2024). Roadmap on Methods and Software for Electronic Structure
Based Simulations in Chemistry and Materials. Electron. Struct..

[7] Cheong P. H.-Y., Legault C. Y., Um J. M., Çelebi-Ölçüm N., Houk K. N. (2011). Quantum Mechanical Investigations of Organocatalysis:
Mechanisms, Reactivities, and Selectivities. Chem. Rev..

[8] Thiel W. (2014). Computational
Catalysis—Past, Present, and Future. Angew. Chem., Int. Ed..

[9] Lam Y., Grayson M. N., Holland M. C., Simon A., Houk K. N. (2016). Theory
and Modeling of Asymmetric Catalytic Reactions. Acc. Chem. Res..

[10] Sameera W. M. C., Maeda S., Morokuma K. (2016). Computational Catalysis
Using the
Artificial Force Induced Reaction Method. Acc.
Chem. Res..

[11] Harvey J. N., Himo F., Maseras F., Perrin L. (2019). Scope and Challenge
of Computational Methods for Studying Mechanism and Reactivity in
Homogeneous Catalysis. ACS Catal..

[12] Foscato M., Jensen V. R. (2020). Automated in Silico
Design of Homogeneous Catalysts. ACS Catal..

[13] Maloney M. P., Stenfors B. A., Helquist P., Norrby P.-O., Wiest O. (2023). Interplay
of Computation and Experiment in Enantioselective Catalysis: Rationalization,
Prediction, and–Correction?. ACS Catal..

[14] Drudis-Solé G., Ujaque G., Maseras F., Lledós A. (2005). A QM/MM Study
of the Asymmetric Dihydroxylation of Terminal Aliphatic *n* -Alkenes with OsO_4_ ·(DHQD)_2_ PYDZ: Enantioselectivity
as a Function of Chain Length. Chem.—Eur.
J..

[15] Maeda S., Ohno K. (2008). Lowest Transition State for the Chirality-Determining Step in Ru­((*R*)-BINAP)-Catalyzed Asymmetric Hydrogenation of Methyl-3-Oxobutanoate. J. Am. Chem. Soc..

[16] Donoghue P. J., Helquist P., Norrby P.-O., Wiest O. (2009). Prediction of Enantioselectivity
in Rhodium Catalyzed Hydrogenations. J. Am.
Chem. Soc..

[17] Hatanaka M., Maeda S., Morokuma K. (2013). Sampling of
Transition States for
Predicting Diastereoselectivity Using Automated Search Method—Aqueous
Lanthanide-Catalyzed Mukaiyama Aldol Reaction. J. Chem. Theory Comput..

[18] Guan Y., Ingman V. M., Rooks B. J., Wheeler S. E. (2018). AARON: An Automated
Reaction Optimizer for New Catalysts. J. Chem.
Theory Comput..

[19] Rosales A. R., Wahlers J., Limé E., Meadows R. E., Leslie K. W., Savin R., Bell F., Hansen E., Helquist P., Munday R. H., Wiest O., Norrby P.-O. (2019). Rapid Virtual Screening
of Enantioselective Catalysts Using CatVS. Nat.
Catal..

[20] Pracht P., Bohle F., Grimme S. (2020). Automated Exploration of the Low-Energy
Chemical Space with Fast Quantum Chemical Methods. Phys. Chem. Chem. Phys..

[21] Pracht P., Grimme S., Bannwarth C., Bohle F., Ehlert S., Feldmann G., Gorges J., Müller M., Neudecker T., Plett C., Spicher S., Steinbach P., Wesołowski P. A., Zeller F. (2024). CREST—A Program for the Exploration
of Low-Energy Molecular Chemical Space. J. Chem.
Phys..

[22] Laplaza R., Wodrich M. D., Corminboeuf C. (2024). Overcoming
the Pitfalls of Computing
Reaction Selectivity from Ensembles of Transition States. J. Phys. Chem. Lett..

[23] Senn H. M., Thiel W. (2009). QM/MM Methods for Biomolecular Systems. Angew.
Chem., Int. Ed..

[24] Chung L. W., Sameera W. M. C., Ramozzi R., Page A. J., Hatanaka M., Petrova G. P., Harris T. V., Li X., Ke Z., Liu F., Li H.-B., Ding L., Morokuma K. (2015). The ONIOM Method and
Its Applications. Chem. Rev..

[25] Grimme S., Bannwarth C., Shushkov P. (2017). A Robust and Accurate Tight-Binding
Quantum Chemical Method for Structures, Vibrational Frequencies, and
Noncovalent Interactions of Large Molecular Systems Parametrized for
All Spd-Block Elements (*Z* = 1–86). J. Chem. Theory Comput..

[26] Bannwarth C., Ehlert S., Grimme S. (2019). GFN2-xTB—An
Accurate and Broadly
Parametrized Self-Consistent Tight-Binding Quantum Chemical Method
with Multipole Electrostatics and Density-Dependent Dispersion Contributions. J. Chem. Theory Comput..

[27] Hatanaka, M. ; Yoshimura, T. ; Maeda, S. Artificial Force-Induced Reaction Method for Systematic Elucidation of Mechanism and Selectivity in Organometallic Reactions. In New Directions in the Modeling of Organometallic Reactions; Lledós, A. , Ujaque, G. , Eds.; Topics in Organometallic Chemistry; Springer International Publishing: Cham, 2020; Vol. 67, pp 57–80. 10.1007/3418_2020_51.

[28] Schlegel H. B. (2003). Exploring
Potential Energy Surfaces for Chemical Reactions: An Overview of Some
Practical Methods. J. Comput. Chem..

[29] Wales, D. Energy Landscapes: Applications to Clusters, Biomolecules and Glasses, 1st ed.; Cambridge University Press, 2001. 10.1017/CBO9780511721724.

[30] Maeda S., Harabuchi Y., Ono Y., Taketsugu T., Morokuma K. (2015). Intrinsic Reaction Coordinate: Calculation, Bifurcation,
and Automated Search. Int. J. Quantum Chem..

[31] Dewyer A. L., Argüelles A. J., Zimmerman P. M. (2018). Methods for Exploring Reaction Space
in Molecular Systems. WIREs Comput. Mol. Sci..

[32] Simm G. N., Vaucher A. C., Reiher M. (2019). Exploration
of Reaction Pathways
and Chemical Transformation Networks. J. Phys.
Chem. A.

[33] Margraf J. T., Jung H., Scheurer C., Reuter K. (2023). Exploring Catalytic
Reaction Networks with Machine Learning. Nat.
Catal..

[34] Schlegel H. B. (2025). Exploring
Potential Energy Surfaces. Pure Appl. Chem..

[35] Maeda S., Harabuchi Y., Takagi M., Taketsugu T., Morokuma K. (2016). Artificial Force Induced
Reaction (AFIR) Method for
Exploring Quantum Chemical Potential Energy Surfaces. Chem. Rec..

[36] Maeda S., Harabuchi Y. (2021). Exploring Paths of Chemical Transformations in Molecular
and Periodic Systems: An Approach Utilizing Force. WIREs Comput. Mol. Sci..

[37] Mita T., Takano H., Hayashi H., Kanna W., Harabuchi Y., Houk K. N., Maeda S. (2022). Prediction of High-Yielding Single-Step
or Cascade Pericyclic Reactions for the Synthesis of Complex Synthetic
Targets. J. Am. Chem. Soc..

[38] Maeda S., Harabuchi Y., Hayashi H., Mita T. (2023). Toward Ab Initio Reaction
Discovery Using the Artificial Force Induced Reaction Method. Annu. Rev. Phys. Chem..

[39] Sumiya Y., Maeda S. (2019). A Reaction Path Network
for Wöhler’s Urea Synthesis. Chem.
Lett..

[40] Sumiya Y., Nagahata Y., Komatsuzaki T., Taketsugu T., Maeda S. (2015). Kinetic Analysis for the Multistep
Profiles of Organic Reactions:
Significance of the Conformational Entropy on the Rate Constants of
the Claisen Rearrangement. J. Phys. Chem. A.

[41] Iwata, S. ; Oki, T. ; Sakaue, S. Rate Constant Matrix Contraction Method for Stiff Master Equations with Detailed Balance. arXiv, 2023. 10.48550/ARXIV.2312.05470.

[42] Ramakrishnan R., Dral P. O., Rupp M., Von Lilienfeld O. A. (2015). Big Data
Meets Quantum Chemistry Approximations: The Δ-Machine Learning
Approach. J. Chem. Theory Comput..

[43] Zheng P., Zubatyuk R., Wu W., Isayev O., Dral P. O. (2021). Artificial
Intelligence-Enhanced Quantum Chemical Method with Broad Applicability. Nat. Commun..

[44] Käser S., Vazquez-Salazar L. I., Meuwly M., Töpfer K. (2023). Neural Network
Potentials for Chemistry: Concepts, Applications and Prospects. Digital Discovery.

[45] Staub R., Gantzer P., Harabuchi Y., Maeda S., Varnek A. (2023). Challenges
for Kinetics Predictions via Neural Network Potentials: A Wilkinson’s
Catalyst Case. Molecules.

[46] Staub R., Harabuchi Y., Seraphim C., Varnek A., Maeda S. (2026). An Accurate
and Efficient Reaction Path Search with Iteratively Trained Neural
Network Potential: Answering the Passerini Mechanism Controversy. J. Chem. Theory Comput..

[47] Tsuji N., Kennemur J. L., Buyck T., Lee S., Prévost S., Kaib P. S. J., Bykov D., Farès C., List B. (2018). Activation of Olefins via Asymmetric Brønsted Acid Catalysis. Science.

[48] Harden I., Neese F., Bistoni G. (2022). An Induced-Fit Model for Asymmetric
Organocatalytic Reactions: A Case Study of the Activation of Olefins *via* Chiral Brønsted Acid Catalysts. Chem. Sci..

[49] Li B., Guan X., Yang S., Zou Y., Liu W., Houk K. N. (2022). Mechanism of the Stereoselective Catalysis of Diels-Alderase
PyrE3 Involved in Pyrroindomycin Biosynthesis. J. Am. Chem. Soc..

[50] Legault, C. Y. CYLview, 1.0b, 2009.

[51] Sumiya Y., Maeda S. (2019). A Reaction Path Network for Wöhler’s Urea Synthesis. Chem. Lett..

[52] Sumiya Y., Maeda S. (2020). Rate Constant Matrix Contraction Method for Systematic Analysis of
Reaction Path Networks. Chem. Lett..

[53] Jacobs R., Morgan D., Attarian S., Meng J., Shen C., Wu Z., Xie C. Y., Yang J. H., Artrith N., Blaiszik B., Ceder G., Choudhary K., Csanyi G., Cubuk E. D., Deng B., Drautz R., Fu X., Godwin J., Honavar V., Isayev O., Johansson A., Kozinsky B., Martiniani S., Ong S. P., Poltavsky I., Schmidt K., Takamoto S., Thompson A. P., Westermayr J., Wood B. M. (2025). A Practical Guide
to Machine Learning Interatomic Potentials
- Status and Future. Curr. Opin. Solid State
Mater. Sci..

[54] Grimme S., Bannwarth C., Shushkov P. (2017). A Robust and Accurate Tight-Binding
Quantum Chemical Method for Structures, Vibrational Frequencies, and
Noncovalent Interactions of Large Molecular Systems Parametrized for
All Spd-Block Elements (*Z* = 1–86). J. Chem. Theory Comput..

[55] Ayala P. Y., Schlegel H. B. (1997). A Combined Method for Determining
Reaction Paths, Minima,
and Transition State Geometries. J. Chem. Phys..

[56] Choi C., Elber R. (1991). Reaction Path Study
of Helix Formation in Tetrapeptides: Effect of
Side Chains. J. Chem. Phys..

[57] Frisch, M. J. ; Trucks, G. W. ; Schlegel, H. B. ; Scuseria, G. E. ; Robb, M. A. ; Cheeseman, J. R. ; Scalmani, G. ; Barone, V. ; Petersson, G. A. ; Nakatsuji, H. ; Li, X. ; Caricato, M. ; Marenich, A. V. ; Bloino, J. ; Janesko, B. G. ; Gomperts, R. ; Mennucci, B. ; Hratchian, H. P. ; Ortiz, J. V. ; Izmaylov, A. F. ; Sonnenberg, J. L. ; Williams; Ding, F. ; Lipparini, F. ; Egidi, F. ; Goings, J. ; Peng, B. ; Petrone, A. ; Henderson, T. ; Ranasinghe, D. ; Zakrzewski, V. G. ; Gao, J. ; Rega, N. ; Zheng, G. ; Liang, W. ; Hada, M. ; Ehara, M. ; Toyota, K. ; Fukuda, R. ; Hasegawa, J. ; Ishida, M. ; Nakajima, T. ; Honda, Y. ; Kitao, O. ; Nakai, H. ; Vreven, T. ; Throssell, K. ; Montgomery, J. A., Jr. ; Peralta, J. E. ; Ogliaro, F. ; Bearpark, M. J. ; Heyd, J. J. ; Brothers, E. N. ; Kudin, K. N. ; Staroverov, V. N. ; Keith, T. A. ; Kobayashi, R. ; Normand, J. ; Raghavachari, K. ; Rendell, A. P. ; Burant, J. C. ; Iyengar, S. S. ; Tomasi, J. ; Cossi, M. ; Millam, J. M. ; Klene, M. ; Adamo, C. ; Cammi, R. ; Ochterski, J. W. ; Martin, R. L. ; Morokuma, K. ; Farkas, O. ; Foresman, J. B. ; Fox, D. J. Gaussian 16, Rev. C.01; Gaussian, 2016.

[58] Chai J.-D., Head-Gordon M. (2008). Long-Range Corrected Hybrid Density Functionals with
Damped Atom-Atom Dispersion Corrections. Phys.
Chem. Chem. Phys..

[59] Tomasi J., Mennucci B., Cammi R. (2005). Quantum Mechanical
Continuum Solvation
Models. Chem. Rev..

[60] Maeda S., Ohno K., Morokuma K. (2013). Systematic
Exploration of the Mechanism
of Chemical Reactions: The Global Reaction Route Mapping (GRRM) Strategy
Using the ADDF and AFIR Methods. Phys. Chem.
Chem. Phys..

