# Supplementary material for: Predicting Enantioselectivity via Kinetic Simulations on Gigantic Reaction Path Networks
Source: ACS Cent Sci. 2026 Mar 30;12(4):524–31. doi: 10.1021/acscentsci.6c00079 (PMC13107212; doi:10.1021/acscentsci.6c00079)
Supplement: Supplementary file 1 [file oc6c00079_si_001.pdf]

Supporting information

Predicting Enantioselectivity via Kinetic Simulations on Gigantic Reaction Path Networks

Yu Harabuchi,[1,2] Ruben Staub,[1] Min Gao,[1] Nobuya Tsuji,[1] Benjamin List,[1,3] Alexandre Varnek,[1,4] and Satoshi Maeda[1,2,5*]

1. Institute for Chemical Reaction Design and Discovery (WPI-ICReDD), Hokkaido University, Kita 21, Nishi 10, Kita-ku, Sapporo, Hokkaido 001-0021, Japan.

2. JST, ERATO Maeda Artificial Intelligence in Chemical Reaction Design and Discovery Project, Kita 10, Nishi 8, Kita-ku, Sapporo, Hokkaido 060-0810, Japan.

3. Max-Planck-Institut fur Kohlenforschung, Mulheim an der Ruhr 45470, Germany.

4. Laboratory of Chemoinformatics, UMR 7140, CNRS, University of Strasbourg, 67081 Strasbourg, France.

5. Department of Chemistry, Faculty of Science, Hokkaido University, Kita 10, Nishi 8, Kita-ku, Sapporo, Hokkaido 060-0810, Japan.

SI1. Computational details for reaction path search using the NNP/AFIR method

An automated reaction path search was performed by the single component artificial force induced reaction (SC-AFIR)[19] method using the GRRM23 program[20] interfaced with the Gaussian 16 program.[21] SC-AFIR is a method to search for many structures and reaction paths systematically by applying artificial forces between fragments automatically defined in the system. In all the calculations, we used the ωB97X-D functional[22], Def2-SVP basis set under the assumption of the PCM, Solvent=CycloHexane (indicated by ωB97X-D/Def2-SVP level). The collision energy parameter (γ) was set to 300 kJ/mol in the SC-AFIR search making the DFT database, and 200 kJ/mol in the SC-AFIR of 6th iteration (as shown in **Table 1**). The SC-AFIR searches started from the 400 randomly generated geometries (using the GRRM program option of NRUN=400). We employed the kinetic-based navigation method[23] to efficiently search for the reaction paths within the kinetically accessible regions. In the kinetic-based navigation, the reaction temperatures were set at 300 K, 333.15 K (60

^{o}C), and 350 K, and a reaction time was set to 2 days (172800seconds), and the initial population of the kinetic simulations is set to 1/400 for 400 random initial geometries. The search was terminated after computing a specific number of paths shown in **Table 1**. To prevent a molecule from moving too far from the reaction center, a weak force with γ = 100/[N*(N–1)/2] kJ/mol was applied to all atom pairs within the reaction center, where N corresponds to the number of atoms in the reaction center (using a GRRM program option of UniversalForceTarget=1-2,6-8,15,29-32,35-38,43,60). The atoms within the reaction center are denoted by a blue shadow in **Figure S2**. During the search, the PriorityPath option in the GRRM program is used to prioritise the exploration of C-O bond generation and dissociation paths (using the GRRM program option of PriorityPath; 2 6 plus; 2 6 minus; END). In the SC-AFIR search and Repath calculations, all the reaction paths are relaxed using the locally updated planes (LUP)method,[24] and the energy maximum point along the paths is used as a TS geometry during the kinetic analyses. In the Repath calculation (7th and 8th iterations), all the obtained LUP paths in the SC-AFIR search (6th iteration) are further relaxed on the original potential surface by eliminating the additional forces. In the 9th iteration, the Gibbs energies were computed on EQs and TSs of the network. The Gibbs energy correction evaluated under the harmonic vibrational and rigid-rotor approximations was applied by setting all frequencies below 100 cm^{-1} to 100 cm^{-1} (except for the imaginary frequency corresponding to the transition state). During the RCMC simulation based on the reaction path network of the 9th iteration, the energies of several geometries (7 EQs and 10 TSs) with unreasonable Hessian eigenvalues were replaced by 2000 kJ/mol with reference to the most stable reactant to avoid the contamination of these geometries during the analyses.

In the analyses, EQs were classified according to their bonding patterns and the absolute configuration in the product region. All 20,913 (7 EQs are excluded from 20,920) EQs were grouped into 74 categories based on bonding patterns and stereochemical conformations at the reaction site. For this classification, only the substrate (atoms 1–26) and the core part of the catalyst (atoms 27–38) were considered when determining the bonding patterns highlighted by red outlines. If an atom within atom 1-38 and another atom in the catalyst skeleton were swapped, they are classified into different groups.

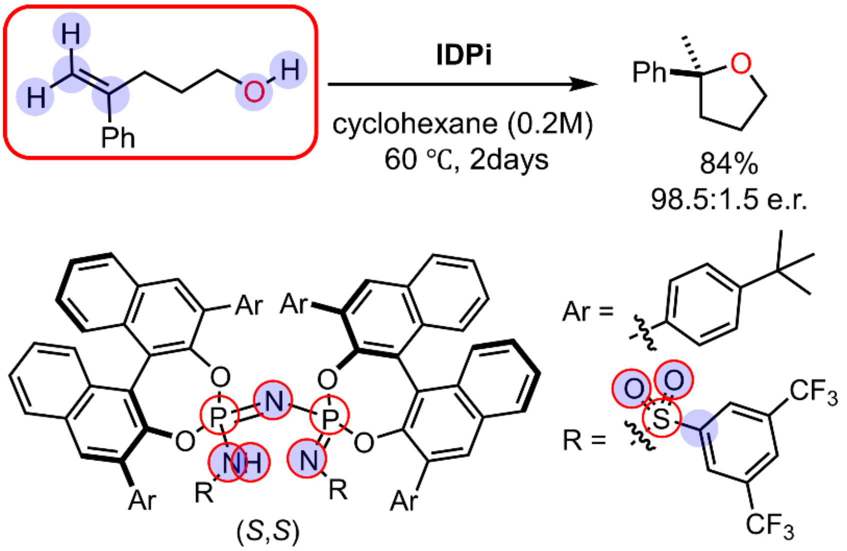

Figure S2. Reaction scheme of the target system. Atoms within the reaction center are indicated by a blue shadow. The atoms considered in the geometry classification are highlighted by red outlines.

	NNP	Def2-SVP	Def2-TZVP
G9<br>EQ236	0.0	0.0	0.0
G9 – G36<br>PT47378	97.5	95.6	99.0
G36<br>EQ8057	77.9	73.3	71.0
G9 – G11<br>PT37289	101.7	98.9	100.1
G9 – G10<br>PT41816	116.4	116.0	117.6
G10<br>EQ3870	−2.0	−0.4	1.1
G11<br>EQ3979	1.0	3.1	0.2
G36 – G11<br>PT44836	92.3	91.2	78.5

Table S3. The comparison of the electronic energy of the intermediate and TS's main reaction pathway with different basis sets. The reference energy is set to the reactant G9, EQ236. NNP: The NNP model in current work; Def2SVP: ωB97X-D/Def2-SVP with PCM

(Solvent = CycloHexane); Def2TZVP: ωB97X-D/Def2-TZVP with PCM (Solvent = CycloHexane).

	The value for the most stable geometry	The ratio of traffic volume > 10^{-5} (%)
G4	4.91×10^{-6} EQ5179	0.0%
G6	1.43×10^{-4} EQ2428	100.0%
G9	2.58×10^{-4} EQ236	99.3%
G10	3.95×10^{-6} EQ3870	0.0%
G11	1.18×10^{-4} EQ3979	100.0%
G12	1.52×10^{-4} EQ4998	99.9%
G15	2.42×10^{-6} EQ9826	0.0%
G18	9.68×10^{-5} EQ2048	100.0%
G19	1.05×10^{-4} EQ20887	98.8%
G29	5.48×10^{-5} EQ10057	61.1%
G35	4.57×10^{-5} EQ18649	100.0%
G36	4.74×10^{-5} EQ8057	99.1%

Table S4. For each group, the traffic volume of the most stable configuration, and the fraction of configurations with traffic volume > 1×10^{-5} are presented.
